# Adaptive fixed-time TSM for uncertain nonlinear dynamical system under unknown disturbance

**DOI:** 10.1371/journal.pone.0304448

**Published:** 2024-08-21

**Authors:** Saim Ahmed, Ahmad Taher Azar, Haoping Wang

**Affiliations:** 1 College of Computer and Information Sciences Prince Sultan University Riyadh, Riyadh, Saudi Arabia; 2 Automated Systems and Soft Computing Lab (ASSCL), Prince Sultan University, Riyadh, Saudi Arabia; 3 Faculty of Computers and Artificial Intelligence, Benha University, Benha, Egypt; 4 School of Automation, Nanjing University of Science and Technology, Nanjing, Jiangsu, China; Lanzhou University of Technology, CHINA

## Abstract

For nonlinear systems subjected to external disturbances, an adaptive terminal sliding mode control (TSM) approach with fixed-time convergence is presented in this paper. The introduction of the fixed-time TSM with the sliding surface and the new Lemma of fixed-time stability are the main topics of discussion. The suggested approach demonstrates quick convergence, smooth and non-singular control input, and stability within a fixed time. Existing fixed-time TSM schemes are often impacted by unknown dynamics such as uncertainty and disturbances. Therefore, the proposed strategy is developed by combining the fixed-time TSM with an adaptive scheme. This adaptive method deals with an uncertain dynamic system when there are external disturbances. The stability of a closed-loop structure in a fixed-time will be shown by the findings of the Lyapunov analysis. Finally, the outcomes of the simulations are shown to evaluate and demonstrate the efficacy of the suggested method. As a result, examples with different cases are provided for a better comparison of suggested and existing control strategies.

## 1. Introduction

Networked systems, robotic arm systems, bio-mathematical systems, renewable energy systems, chaotic systems, and servo control systems are just a few examples of the numerous applications in applied mathematics that commonly deal with nonlinear systems influenced by outside perturbations [1–5]. Therefore, the performance and stability of these systems are commonly affected by uncertainties, nonlinearities, and disturbances [6–10]. To cope with these issues, researchers have developed various control strategies, including adaptive control and sliding mode control [11]. Thus, adaptive control and sliding mode control schemes improve transient performance and provide robustness for the entire dynamical system by combining the advantages of these control techniques [12]. As a result, a robust control approach for nonlinear perturbed dynamical system is obtained as well as adaptive scheme deals with unknown uncertain dynamics [13].

Due to its ability to effectively manage uncertainties and disturbances, the sliding mode scheme has seen extensive use in the development of nonlinear control systems, resulting in overall system robustness [14–16]. Nevertheless, this method has an issue with chattering occurring in the control input. Chattering can cause undesired noise to be produced inside the system, which could cause performance instability [17]. Therefore, employing an integral sliding mode, incorporating a boundary layer, or applying a saturation function are some of the approaches that have been proposed to reduce chattering [13, 18, 19]. This scheme has also been applied to various applications such as robotic manipulator [20], unmanned aerial vehicle (UAV) [21], suspension systems [22, 23], biomathematical models [24], etc. Furthermore, finite-time terminal nonsingular (FTNSMC) has been developed in recent years to achieve superior tracking, nonsingularity, and rapid state convergence [25, 26]. Nonetheless, the convergence performance of finite-time approach is considerably affected by the beginning values of the states [27]. Therefore, a substitute that may be utilized to calculate the convergence time that is not dependent on the initial values is a fixed-time control technique [28].

By adjusting the controller parameters according to the present scenario of the system, the method is known as adaptive control [29]. It is a popular control strategy that is commonly used to deal with unknown parameters in nonlinear systems. Therefore, by adjusting the controller’s parameters in real time to compensate for the impacts of nonlinearity, uncertainty, as well as disturbances, this method can enhance a system’s performance and stability [30]. Furthermore, this control strategy is widely used to regulate the dynamics of linear and nonlinear systems [29]. Several classical and advanced control methods have been found to combine well with adaptive and TSM controllers. The new hybrid algorithm with PID-TSM has been proposed to significantly reduce vibration in active suspension systems [22]. An adaptive differential evolution tuned hybrid fuzzy PD-PI controller for power system automatic generation control has been proposed. Evaluations via simulations, stability analysis, and hardware-in-the-Loop real-time validation confirm its effectiveness [31, 32]. An adaptive fixed-time TSM has been developed for the application of Euler-Lagrange systems under unknown dynamics [13]. A TSM based time delay estimation scheme has been designed for the unknown dynamics of robotic manipulator [20]. Moreover, model reference adaptive H∞ control scheme has been proposed to control dynamics of the robotic exoskeleton [33]. An innovative adaptive MPPT approach is proposed for photovoltaic system, combining the incremental conductance method with a fuzzy self-tuning PID controller. Evaluations demonstrate superior efficiency (up to 99.80%) and precise control compared to conventional MPPT techniques under diverse climate scenarios [34]. Hence, the combined application of adaptive and sliding mode control strategies has emerged as a powerful approach for nonlinear system control. This synergistic integration offers several advantages, including enhanced robustness to system uncertainties, improved tracking performance, expedited convergence to desired states, the elimination of non-smooth control inputs, and the capability to handle systems with unknown parameters [35].

Motivated by the schemes mentioned in literature, such as adaptive control and fixed-time TSM, this work is to design the new adaptive fixed sliding mode control scheme. Fixed-time control, which is not affected by the initial conditions of the states, has been employed in this work as the convergence speed of a finite-time scheme varies as the initial value changes. Moreover, an adaptive scheme is used to control the unknown dynamics in the system. Therefore, a new adaptive sliding mode control is proposed that offers to handle the control of a class of nonlinear systems affected by external disturbance with fixed-time convergence, better tracking, and transient responses, as well as obtaining a robust performance closed-loop system. The key points are given as

1. The new fixed time sliding mode scheme is designed to achieve fast convergence within a fixed time.
2. The adaptive law is combined with fixed-time TSM and is used to deal with the unknown bounded dynamics.
3. The investigation of overall system stability with fixed-time convergence is carried out by the Lyapunov synthesis.
4. The comparative simulation analyses are provided to validate the efficacy of the proposed scheme.

The outline of this paper is arranged as follows: The proposed fixed-time TSM scheme with closed-loop stability analyses is given in Section 2. The key findings, together with the recommended adaptive sliding mode control strategy and overall stability analysis, are provided in Section 3. Numerical results of the computational simulation are presented in Section 4. The evaluation and discussion are given in Section 5. In the end, conclusion is given in Section 6.

## 2. Proposed fixed-time sliding control method

In this section, the designed time-varying fixed-time sliding surface is given. Then, the proposed control fixed-time sliding mode scheme is developed, and a closed-loop system is provided using a nonlinear dynamical system with external disturbances given as

$$\begin{array}{c} \begin{array}{c} {\dot{X}}_{1}=X_{2}\\ {\dot{X}}_{2}=f\left(X\right)+g\left(X\right)u\left(t\right)+F\left(t\right)+d\left(t\right) \end{array} \end{array}$$ (1)

where $X={\left[X_{1},X_{2}\right]}^{T}\in R$, f(X) and g(X)≠0 are nonlinear functions, F(t)∈R is uncertainty, d(t)∈R is the unknown disturbance, and u(t)∈R is the control input.

The tracking error will be utilized in the suggested control method and stability analysis; therefore, it is given in (1) as follows:

$$\begin{array}{c} \begin{array}{c} \ddot{\varepsilon }=f\left(X\right)+g\left(X\right)u\left(t\right)+D\left(t\right)-{\ddot{X}}_{d} \end{array} \end{array}$$ (2)

where $\varepsilon =X_{1}-X_{d}$ and $X_{d}$ is the reference input, and D(t)=F(t)+d(t) with the bounded condition |D(t)|≤κ, *κ* > 0 is unknown constant.

**Lemma 1** [36]: The following nonlinear dynamics are considered:

$$\begin{array}{c} \dot{Y}\left(t\right)=f\left(t,Y\right),\;\;\;Y\left(0\right)=Y_{0} \end{array}$$ (3)

where f(t,Y) denotes the continuous function. Thus, the Lyapunov function V(Y) that holds:

$$\begin{array}{c} \dot{V}\left(Y\right)\leq -{\bar{p}}_{1}V^{{\bar{q}}_{1}}\left(Y\right)-{\bar{p}}_{2}V{\left(Y\right)}^{{\bar{q}}_{2}}-{\bar{p}}_{3}V\left(Y\right) \end{array}$$

with V(Y)=0⇒Y=0, ${\bar{p}}_{1},\;{\bar{p}}_{2},\;{\bar{p}}_{3}>0,\;{\bar{q}}_{1}>1\;\;\;and\;\;{\bar{q}}_{2}\in \left(0,1\right)$. The settling time is computed as

$$\begin{array}{c} T\leq \frac{1}{{\bar{p}}_{3}\left({\bar{q}}_{1}-1\right)}\text{ln}\left(1+\frac{{\bar{p}}_{3}}{{\bar{p}}_{1}}\right)+\frac{1}{{\bar{p}}_{3}\left(1-{\bar{q}}_{2}\right)}\text{ln}\left(1+\frac{{\bar{p}}_{3}}{{\bar{p}}_{2}}\right). \end{array}$$ (4)

### 2.1 Design of sliding surface

The design of a fixed-time sliding surface is given in this subsection. It offers robust performance and precise trajectory tracking for nonlinear systems in fixed-time. The proposed sliding surface is provided as

$$\begin{array}{c} \sigma ={\left({\left|\varepsilon \right|}^{\alpha_{2}}sign\left(\varepsilon \right)\right)}^{1/\alpha_{2}}+{\left(\frac{\dot{\varepsilon }+\gamma_{3}|\varepsilon |sign\left(\varepsilon \right)}{\gamma_{2}+\gamma_{1}{\left|\varepsilon \right|}^{\alpha_{1}-\alpha_{2}}}\right)}^{1/\alpha_{2}} \end{array}$$ (5)

where *γ*_1_, *γ*_2_, *γ*_3_ are positive constants, *α*_1_ > 1 and *α*_2_ ∈ (0, 1). For brevity, (5) can be rewritten as

$$\begin{array}{c} \sigma ={\left({\left|\varepsilon \right|}^{\alpha_{2}}sign\left(\varepsilon \right)\right)}^{1/\alpha_{2}}+{\left(\eta \left(\varepsilon \right)(\dot{\varepsilon }+\gamma_{3}|\varepsilon |sign\left(\varepsilon \right))\right)}^{1/\alpha_{2}} \end{array}$$ (6)

with $\eta \left(\varepsilon \right)=\frac{1}{\gamma_{2}+\gamma_{1}{\left|\varepsilon \right|}^{\alpha_{1}-\alpha_{2}}}$.

From (6), $\dot{\sigma }$ can be computed as

$$\begin{array}{c} \begin{array}{c} \dot{\sigma }={\left({\left|\varepsilon \right|}^{\alpha_{2}}sign\left(\varepsilon \right)\right)}^{1/\alpha_{2}-1}{\left|\varepsilon \right|}^{\alpha_{2}-1}\dot{\varepsilon }\\ +\frac{1}{\alpha_{2}}{\left(\eta \left(\varepsilon \right)(\dot{\varepsilon }+\gamma_{3}|\varepsilon |sign\left(\varepsilon \right))\right)}^{1/\alpha_{2}-1}\\ \times [\begin{array}{c} \frac{\left(\gamma_{2}+\gamma_{1}{\left|\varepsilon \right|}^{\alpha_{1}-\alpha_{2}}\right)\left(\ddot{\varepsilon }+\gamma_{3}\dot{\varepsilon }\right)}{{\left(\gamma_{2}+\gamma_{1}{\left|\varepsilon \right|}^{\alpha_{1}-\alpha_{2}}\right)}^{2}}\\ -\frac{\gamma_{1}\left(\alpha_{1}-\alpha_{2}\right){\left|\varepsilon \right|}^{\alpha_{1}-\alpha_{2}-1}\dot{\varepsilon }\left(\dot{\varepsilon }+\gamma_{3}|\varepsilon |sign\left(\varepsilon \right)\right)}{{\left(\gamma_{2}+\gamma_{1}{\left|\varepsilon \right|}^{\alpha_{1}-\alpha_{2}}\right)}^{2}} \end{array}] \end{array} \end{array}$$ (7)

Then (7) can be expressed as

$$\begin{array}{c} \begin{array}{c} \dot{\sigma }={\left({\left|\varepsilon \right|}^{\alpha_{2}}sign\left(\varepsilon \right)\right)}^{1/\alpha_{2}-1}{\left|\varepsilon \right|}^{\alpha_{2}-1}\dot{\varepsilon }\\ +\frac{1}{\alpha_{2}}{\left(\eta \left(\varepsilon \right)(\dot{\varepsilon }+\gamma_{3}|\varepsilon |sign\left(\varepsilon \right))\right)}^{1/\alpha_{2}-1}\\ \times [\begin{array}{c} \eta \left(\varepsilon \right)\left(\ddot{\varepsilon }+\gamma_{3}\dot{\varepsilon }\right)\\ -\gamma_{1}\left(\alpha_{1}-\alpha_{2}\right){\left|\varepsilon \right|}^{\alpha_{1}-\alpha_{2}-1}\eta {\left(\varepsilon \right)}^{2}\dot{\varepsilon }\left(\dot{\varepsilon }+\gamma_{3}|\varepsilon |sign\left(\varepsilon \right)\right) \end{array}] \end{array} \end{array}$$ (8)

Tracking error (2) substituted into (8), we can have

$$\begin{array}{c} \begin{array}{c} \dot{\sigma }={\left({\left|\varepsilon \right|}^{\alpha_{2}}sign\left(\varepsilon \right)\right)}^{1/\alpha_{2}-1}{\left|\varepsilon \right|}^{\alpha_{2}-1}\dot{\varepsilon }\\ +\frac{1}{\alpha_{2}}{\left(\eta \left(\varepsilon \right)(\dot{\varepsilon }+\gamma_{3}|\varepsilon |sign\left(\varepsilon \right))\right)}^{1/\alpha_{2}-1}\\ \times [\begin{array}{c} \eta \left(\varepsilon \right)(f\left(X\right)+g\left(X\right)u\left(t\right)+D\left(t\right)-{\ddot{X}}_{d}+\gamma_{3}\dot{\varepsilon })\\ -\gamma_{1}\left(\alpha_{1}-\alpha_{2}\right){\left|\varepsilon \right|}^{\alpha_{1}-\alpha_{2}-1}\eta {\left(\varepsilon \right)}^{2}\dot{\varepsilon }\left(\dot{\varepsilon }+\gamma_{3}|\varepsilon |sign\left(\varepsilon \right)\right) \end{array}] \end{array} \end{array}$$ (9)

Now that the sliding manifold design has been finalized, the next phase will be designing the proposed scheme using fixed-time terminal sliding mode control for the nonlinear system to achieve robust tracking and fast convergence despite dealing with external disturbances.

### 2.2 Design fixed-time control scheme

The following formulation (*u*(*t*) = *u*_1_(*t*) + *u*_2_(*t*)) of the suggested scheme using fixed-time TSM can be used to effectively control the nonlinear system when bounded disturbances are present

$$\begin{array}{c} \begin{array}{c} u_{1}\left(t\right)=-g^{-1}\left(X\right)(f\left(X\right)-{\ddot{X}}_{d}+\kappa sign\left(\sigma \right)+\gamma_{3}\dot{\varepsilon })\\ +\frac{g^{-1}\left(X\right)}{\eta (\varepsilon )}(\gamma_{1}\left(\alpha_{1}-\alpha_{2}\right){\left|\varepsilon \right|}^{\alpha_{1}-\alpha_{2}-1}\eta {\left(\varepsilon \right)}^{2}\dot{\varepsilon }\left(\dot{\varepsilon }+\gamma_{3}|\varepsilon |sign\left(\varepsilon \right)\right))\\ \begin{array}{c} -\frac{g^{-1}\left(X\right)\gamma_{2}}{\eta (\varepsilon )}\eta {\left(\varepsilon \right)}^{1-1/\alpha_{2}}{\left(\dot{\varepsilon }+\gamma_{3}|\varepsilon |sign\left(\varepsilon \right)\right)}^{1-1/\alpha_{2}}\\ \times {\left({\left|\varepsilon \right|}^{\alpha_{2}}sign\left(\varepsilon \right)\right)}^{1/\alpha_{2}-1}{\left|\varepsilon \right|}^{\alpha_{2}-1}\dot{\varepsilon } \end{array} \end{array} \end{array}$$ (10)

and

$$\begin{array}{c} \begin{array}{c} u_{2}\left(t\right)=-\frac{g^{-1}\left(X\right)\gamma_{2}}{\eta (\varepsilon )}\eta {\left(\varepsilon \right)}^{1-1/\alpha_{2}}\\ \times (\xi_{1}{\left|\sigma \right|}^{\beta_{1}}sign\left(\sigma \right)+\xi_{2}{\left|\sigma \right|}^{\beta_{2}}sign\left(\sigma \right)+\xi_{3}|\sigma |sign\left(\sigma \right)) \end{array} \end{array}$$ (11)

where *ξ*_1_, *ξ*_2_ and *ξ*_3_ are positive constants, *β*_1_ > 1 and *β*_2_ ∈ (0, 1). From (10) and (11), *u*(*t*) substituted into (9), we can obtain

$$\begin{array}{c} \begin{array}{c} \dot{\sigma }=-{\left(\eta \left(\varepsilon \right)\dot{\varepsilon }\right)}^{1/\alpha_{2}-1}\\ \times [\begin{array}{c} \eta {\left(\varepsilon \right)}^{1-1/\alpha_{2}}(\xi_{1}{\left|\sigma \right|}^{\beta_{1}}sign\left(\sigma \right)+\xi_{2}{\left|\sigma \right|}^{\beta_{2}}sign\left(\sigma \right)+\xi_{3}|\sigma |sign\left(\sigma \right))\\ +\frac{1}{\alpha_{2}}\eta \left(\varepsilon \right)\left(-\kappa sign\left(\sigma \right)+D\left(t\right)\right) \end{array}] \end{array} \end{array}$$ (12)

The design of control method is completed, now the stability analysis of the proposed scheme will be given in next subsection.

### 2.3 Stability analysis

Fixed-time stability investigations of the suggested control scheme have been carried out in this subsection. Moreover, the settling time is computed using the corresponding Lemma 1. The following Lyapunov function equation is selected for the stability analysis of tracking error

$$\begin{array}{c} V_{1}=\frac{1}{2}\varepsilon^{2} \end{array}$$ (13)

The ${\dot{V}}_{1}$ is obtained as

$$\begin{array}{c} {\dot{V}}_{1}=\varepsilon \dot{\varepsilon } \end{array}$$ (14)

The expression from (5) is achieved as $\dot{\varepsilon }=-\gamma_{1}{\left|\varepsilon \right|}^{\alpha_{1}}sign\left(\varepsilon \right)-\gamma_{2}{\left|\varepsilon \right|}^{\alpha_{2}}sign\left(\varepsilon \right)-\gamma_{3}|\varepsilon |sign\left(\varepsilon \right)$ when *σ* = 0. So, the following equation is obtained by putting $\dot{\varepsilon }$ into (14) as

$$\begin{array}{c} {\dot{V}}_{1}=\varepsilon \{-\gamma_{1}{\left|\varepsilon \right|}^{\alpha_{1}}sign\left(\varepsilon \right)-\gamma_{2}{\left|\varepsilon \right|}^{\alpha_{2}}sign\left(\varepsilon \right)-\gamma_{3}|\varepsilon |sign\left(\varepsilon \right)\} \end{array}$$ (15)

$$\begin{array}{c} {\dot{V}}_{1}=-\gamma_{1}{\left|\varepsilon \right|}^{\alpha_{1}+1}-\gamma_{2}{\left|\varepsilon \right|}^{\alpha_{2}+1}-\gamma_{3}{\left|\varepsilon \right|}^{2} \end{array}$$ (16)

The above expression can be written as

$$\begin{array}{c} \begin{array}{c} {\dot{V}}_{1}\leq -\gamma_{1}{\left(2V_{1}\right)}^{\frac{\alpha_{1}+1}{2}}-\gamma_{2}{\left(2V_{1}\right)}^{\frac{\alpha_{2}+1}{2}}-\gamma_{3}2V_{1}\\ \leq -\gamma_{1}2^{\frac{\alpha_{1}+1}{2}}{V_{1}}^{\frac{\alpha_{1}+1}{2}}-\gamma_{2}2^{\frac{\alpha_{2}+1}{2}}{V_{1}}^{\frac{\alpha_{2}+1}{2}}-\gamma_{3}2V_{1} \end{array} \end{array}$$ (17)

This expression represents an equation that converges in a fixed amount of time, hence Lemma 1 can be used to obtain the convergence time $T_{\varepsilon }$ as

$$\begin{array}{c} T_{\varepsilon }\leq \frac{1}{2\gamma_{3}\left(\frac{\alpha_{1}+1}{2}-1\right)}\text{ln}\left(1+\frac{2\gamma_{3}}{2^{\frac{\alpha_{1}+1}{2}}\gamma_{1}}\right)+\frac{1}{2\gamma_{3}\left(1-\frac{\alpha_{2}+1}{2}\right)}\text{ln}\left(1+\frac{2\gamma_{3}}{2^{\frac{\alpha_{2}+1}{2}}\gamma_{2}}\right). \end{array}$$ (18)

The fixed-time stability of a closed-loop nonlinear system will now be investigated in Theorem 1 by applying the Lyapunov theorem.

**Theorem 1:** By considering the nonlinear system as presented in (1), the sliding surface discussed in (5), and the designed control scheme provided in (10)-(11), then it becomes attainable for the nonlinear dynamics to converge states within a fixed time, assuming condition of bounded disturbance is known.

*Proof:* The candidate for the Lyapunov function is selected as:

$$\begin{array}{c} V_{2}=\frac{1}{2}\sigma^{2} \end{array}$$ (19)

The ${\dot{V}}_{2}$ can be obtained as

$$\begin{array}{c} {\dot{V}}_{2}=\sigma \;\dot{\sigma } \end{array}$$ (20)

From (12), $\dot{\sigma }$ is putting into (20), we can obtain

$$\begin{array}{c} {\dot{V}}_{2}=\sigma \{\begin{array}{c} -{\left(\eta \left(\varepsilon \right)\dot{\varepsilon }\right)}^{1/\alpha_{2}-1}\\ \times [\begin{array}{c} \eta {\left(\varepsilon \right)}^{1-1/\alpha_{2}}(\begin{array}{c} \xi_{1}{\left|\sigma \right|}^{\beta_{1}}sign\left(\sigma \right)+\xi_{2}{\left|\sigma \right|}^{\beta_{2}}sign\left(\sigma \right)\\ +\xi_{3}|\sigma |sign\left(\sigma \right) \end{array})\\ +\frac{1}{\alpha_{2}}\eta \left(\varepsilon \right)\left(-\kappa sign\left(\sigma \right)+D\left(t\right)\right) \end{array}] \end{array}\} \end{array}$$ (21)

By using bounded condition of disturbance to simplify the preceding expression, one can derive

$$\begin{array}{c} {\dot{V}}_{2}\leq -{\dot{\varepsilon }}^{1/\alpha_{2}-1}(\xi_{1}{\left|\sigma \right|}^{\beta_{1}+1}+\xi_{2}{\left|\sigma \right|}^{\beta_{2}+1}+\xi_{3}{\left|\sigma \right|}^{2}) \end{array}$$ (22)

The following are two potential scenarios: i) ${\dot{\varepsilon }}^{1/\alpha_{2}-1}>0\;\;\;if\;\;\;\dot{\varepsilon }\neq 0$, and ii) ${\dot{\varepsilon }}^{1/\alpha_{2}-1}=0\;\;\;if\;\;\dot{\varepsilon }=0$. The Lyapunov function exhibits fixed-time stability when the following condition is met: ${\dot{\varepsilon }}^{1/\alpha_{2}-1}>0$ for $\dot{\varepsilon }\neq 0$. If $\dot{\varepsilon }=0$, substitution of *u*(*t*) from (10)-(11) into (2) yields

$$\begin{array}{c} \ddot{\varepsilon }=-\kappa sign\left(\sigma \right)+D\left(t\right)-\frac{\gamma_{2}}{\eta (\varepsilon )}\eta {\left(\varepsilon \right)}^{1-1/\alpha_{2}}\times (\xi_{1}{\left|\sigma \right|}^{\beta_{1}}sign\left(\sigma \right)+\xi_{2}{\left|\sigma \right|}^{\beta_{2}}sign\left(\sigma \right)+\xi_{3}|\sigma |sign\left(\sigma \right)) \end{array}$$

It is obvious that for *σ* > 0 then $\ddot{\varepsilon }<0$, and for *σ* < 0 then $\ddot{\varepsilon }>0$. This indicates that $\dot{\varepsilon }=0$ is unable to be an attractor. Consequently, no trajectory can stay on $\dot{\varepsilon }=0$; all trajectories will cross $\dot{\varepsilon }=0$ and arrive at the sliding surface within a fixed amount of time.

$$\begin{array}{c} \begin{array}{c} {\dot{V}}_{2}\leq -{\dot{\varepsilon }}^{1/\alpha_{2}-1}(\xi_{1}{\left(2V_{2}\right)}^{\frac{\beta_{1}+1}{2}}+\xi_{2}{\left(2V_{2}\right)}^{\frac{\beta_{2}+1}{2}}+\xi_{3}(2V_{2}))\\ \leq -{\dot{\varepsilon }}^{1/\alpha_{2}-1}(\xi_{1}2^{\frac{\beta_{1}+1}{2}}{V_{2}}^{\frac{\beta_{1}+1}{2}}+\xi_{2}2^{\frac{\beta_{2}+1}{2}}{V_{2}}^{\frac{\beta_{2}+1}{2}}+2\xi_{3}V_{2}) \end{array} \end{array}$$ (23)

According to Lemma 1, the fixed settling time $T_{sn}$ is formulated as

$$\begin{array}{c} T_{sn}\leq \frac{1}{2\xi_{3}\left(\frac{\beta_{1}+1}{2}-1\right)}\text{ln}\left(1+\frac{2\xi_{3}}{2^{\frac{\beta_{1}+1}{2}}\xi_{1}}\right)+\frac{1}{2\xi_{3}\left(1-\frac{\beta_{2}+1}{2}\right)}\text{ln}\left(1+\frac{2\xi_{3}}{2^{\frac{\beta_{2}+1}{2}}\xi_{2}}\right) \end{array}$$ (24)

Hence, within a specified duration, the system’s states attain the value of *σ*. According to Lemma 1, the fixed settling time $T_{sn}$ can easily be calculated, and then total time can be obtained as $T_{a}=T_{sn}+T_{\varepsilon }$.

## 3. Proposed adaptive fixed-time TSM scheme

The following scheme provides a thorough explanation of the proposed control input (*u*(*t*) = *u*_2_(*t*) + *u*_3_(*t*)) and provides an adaptive law with TSM to handle the uncertain dynamics caused by an unknown disturbance.

$$\begin{array}{c} \begin{array}{c} u_{3}\left(t\right)=-g^{-1}\left(X\right)(f\left(X\right)-{\ddot{X}}_{d}+\hat{\kappa }sign\left(\sigma \right)+\gamma_{3}\dot{\varepsilon })\\ +\frac{g^{-1}\left(X\right)}{\eta (\varepsilon )}(\gamma_{1}\left(\alpha_{1}-\alpha_{2}\right){\left|\varepsilon \right|}^{\alpha_{1}-\alpha_{2}-1}\eta {\left(\varepsilon \right)}^{2}\dot{\varepsilon }\left(\dot{\varepsilon }+\gamma_{3}|\varepsilon |sign\left(\varepsilon \right)\right))\\ -\frac{g^{-1}\left(X\right)\gamma_{2}}{\eta (\varepsilon )}\eta {\left(\varepsilon \right)}^{1-1/\alpha_{2}}{\left(\dot{\varepsilon }+\gamma_{3}|\varepsilon |sign\left(\varepsilon \right)\right)}^{1-1/\alpha_{2}}\\ \times {\left({\left|\varepsilon \right|}^{\alpha_{2}}sign\left(\varepsilon \right)\right)}^{1/\alpha_{2}-1}{\left|\varepsilon \right|}^{\alpha_{2}-1}\dot{\varepsilon } \end{array} \end{array}$$ (25)

whereas $\hat{\kappa }$ is the estimation of *κ*, *u*_2_(*t*) is similar to (11). The following adaptive law is given to compensate the unknown dynamics:

$$\begin{array}{c} \dot{\hat{\kappa }}=\frac{\upsilon }{\alpha_{2}}\eta {\left(\varepsilon \right)}^{1/\alpha_{2}}{\dot{\varepsilon }}^{1/\alpha_{2}-1}|\sigma | \end{array}$$ (26)

where *υ* is known + *ve* constant.

*Remark 1.* Eq (1), when applied, provides a successful solution to the compensation of uncertain dynamics. As a result, the proposed method is used to attain tracking performance in nonlinear systems while disturbances are present.

By putting *u*_2_(*t*) and *u*_3_(*t*) into (9), we can get

$$\begin{array}{c} \begin{array}{c} \dot{\sigma }=-{\left(\eta \left(\varepsilon \right)\dot{\varepsilon }\right)}^{1/\alpha_{2}-1}\\ \times [\begin{array}{c} \eta {\left(\varepsilon \right)}^{1-1/\alpha_{2}}(\begin{array}{c} \xi_{1}{\left|\sigma \right|}^{\beta_{1}}sign\left(\sigma \right)+\xi_{2}{\left|\sigma \right|}^{\beta_{2}}sign\left(\sigma \right)\\ +\xi_{3}|\sigma |sign\left(\sigma \right) \end{array})\\ +\frac{1}{\alpha_{2}}\eta \left(\varepsilon \right)\left(-\hat{\kappa }sign\left(\sigma \right)+D\left(t\right)\right) \end{array}] \end{array} \end{array}$$ (27)

**Theorem 2:** This discussion pertains to the nonlinear system (1) and its susceptibility to various challenges, such as external disturbance. The sliding surface (5), the proposed control approach (11) and (25), and the adaptive control law (26), will cause the required state convergence within a fixed time.

*Proof:* The appropriate Lyapunov function is given as

$$\begin{array}{c} V_{3}=\frac{1}{2}\sigma^{2}+\frac{1}{2\upsilon }{\tilde{\kappa }}^{2} \end{array}$$ (28)

where $\tilde{\kappa }=\hat{\kappa }-\kappa$ is the estimation error.

The ${\dot{V}}_{3}$ is computed as

$$\begin{array}{c} {\dot{V}}_{3}=\sigma \;\dot{\sigma }+\frac{1}{\upsilon }\;\tilde{\kappa }\;\dot{\hat{\kappa }} \end{array}$$ (29)

By substitution of $\dot{\sigma }$ given in Eq (27) in (29), Eq (30) can be constructed as follows

$$\begin{array}{c} \begin{array}{c} {\dot{V}}_{3}=\sigma \{-{\left(\eta \left(\varepsilon \right)\dot{\varepsilon }\right)}^{1/\alpha_{2}-1}[\begin{array}{c} \eta {\left(\varepsilon \right)}^{1-1/\alpha_{2}}(\begin{array}{c} \xi_{1}{\left|\sigma \right|}^{\beta_{1}}sign\left(\sigma \right)\\ +\xi_{2}{\left|\sigma \right|}^{\beta_{2}}sign\left(\sigma \right)\\ +\xi_{3}|\sigma |sign\left(\sigma \right) \end{array})\\ +\frac{1}{\alpha_{2}}\eta \left(\varepsilon \right)\left(-\hat{\kappa }sign\left(\sigma \right)+D\left(t\right)\right) \end{array}]\}\\ +\frac{1}{\upsilon }\tilde{\kappa }\dot{\hat{\kappa }} \end{array} \end{array}$$ (30)

Considering the bounded disturbance condition and adaptive rule Eq (26), it is feasible to describe Eq (30) as

$$\begin{array}{c} \begin{array}{c} {\dot{V}}_{3}\leq -{\dot{\varepsilon }}^{1/\alpha_{2}-1}(\xi_{1}{\left|\sigma \right|}^{\beta_{1}+1}+\xi_{2}{\left|\sigma \right|}^{\beta_{2}+1}+\xi_{3}{\left|\sigma \right|}^{2})\\ -\frac{1}{\alpha_{2}}{\left(\eta \left(\varepsilon \right)\dot{\varepsilon }\right)}^{1/\alpha_{2}-1}\eta \left(\varepsilon \right)\tilde{\kappa }|\sigma |+\frac{1}{\upsilon }\tilde{\kappa }\dot{\hat{\kappa }}\\ \leq -{\dot{\varepsilon }}^{1/\alpha_{2}-1}(\xi_{1}{\left|\sigma \right|}^{\beta_{1}+1}+\xi_{2}{\left|\sigma \right|}^{\beta_{2}+1}+\xi_{3}{\left|\sigma \right|}^{2}) \end{array} \end{array}$$ (31)

To calculate the settling time, first the above equation is written as

$$\begin{array}{c} \begin{array}{c} {\dot{V}}_{3}\leq -{\dot{\varepsilon }}^{1/\alpha_{2}-1}[\begin{array}{c} \xi_{1}{\left(2(V_{3}-\frac{1}{2\upsilon }{\tilde{\kappa }}^{2})\right)}^{\frac{\beta_{1}+1}{2}}\\ +\xi_{2}{\left(2(V_{3}-\frac{1}{2\upsilon }{\tilde{\kappa }}^{2})\right)}^{\frac{\beta_{2}+1}{2}}\\ +\xi_{3}(2(V_{3}-\frac{1}{2\upsilon }{\tilde{\kappa }}^{2})) \end{array}]\\ =-{\dot{\varepsilon }}^{1/\alpha_{2}-1}[\begin{array}{c} \xi_{1}2^{\frac{\beta_{1}+1}{2}}{\left(1-\frac{1}{2\upsilon V_{3}}{\tilde{\kappa }}^{2}\right)}^{\frac{\beta_{1}+1}{2}}{V_{3}}^{\frac{\beta_{1}+1}{2}}\\ +\xi_{2}2^{\frac{\beta_{2}+1}{2}}{\left(1-\frac{1}{2\upsilon V_{3}}{\tilde{\kappa }}^{2}\right)}^{\frac{\beta_{2}+1}{2}}{V_{3}}^{\frac{\beta_{2}+1}{2}}\\ +\xi_{3}2(1-\frac{1}{2\upsilon V_{3}}{\tilde{\kappa }}^{2})V_{3} \end{array}] \end{array} \end{array}$$ (32)

Then Eq (33) is obtained as

$$\begin{array}{c} {\dot{V}}_{3}\leq -{\dot{\varepsilon }}^{1/\alpha_{2}-1}[\theta_{1}{V_{3}}^{\frac{\beta_{1}+1}{2}}+\theta_{2}{V_{3}}^{\frac{\beta_{2}+1}{2}}+\theta_{3}V_{3}] \end{array}$$ (33)

where $\theta_{1}=\xi_{1}2^{\frac{\beta_{1}+1}{2}}{\left(1-\frac{1}{2\upsilon V_{3}}{\tilde{\kappa }}^{2}\right)}^{\frac{\beta_{1}+1}{2}},\;\;\theta_{2}=\xi_{2}2^{\frac{\beta_{2}+1}{2}}{\left(1-\frac{1}{2\upsilon V_{3}}{\tilde{\kappa }}^{2}\right)}^{\frac{\beta_{2}+1}{2}},\;\;\theta_{3}=\xi_{3}2(1-\frac{1}{2\upsilon V_{3}}{\tilde{\kappa }}^{2})$.

Thus, the settling time $T_{sa}$ is computed by Lemma 1 as

$$\begin{array}{c} T_{sa}\leq \frac{1}{\theta_{3}\left(\frac{\beta_{1}+1}{2}-1\right)}\text{ln}\left(1+\frac{\theta_{3}}{\theta_{1}}\right)+\frac{1}{\theta_{3}\left(1-\frac{\beta_{2}+1}{2}\right)}\text{ln}\left(1+\frac{\theta_{3}}{\theta_{2}}\right) \end{array}$$ (34)

Using $T_{b}=T_{sa}+T_{\varepsilon }$, one may find the equation for the total settling time. As a result, the dynamical system is used to precisely control the system’s states and maintain overall stability over a fixed period of time.

**Fig 1:**
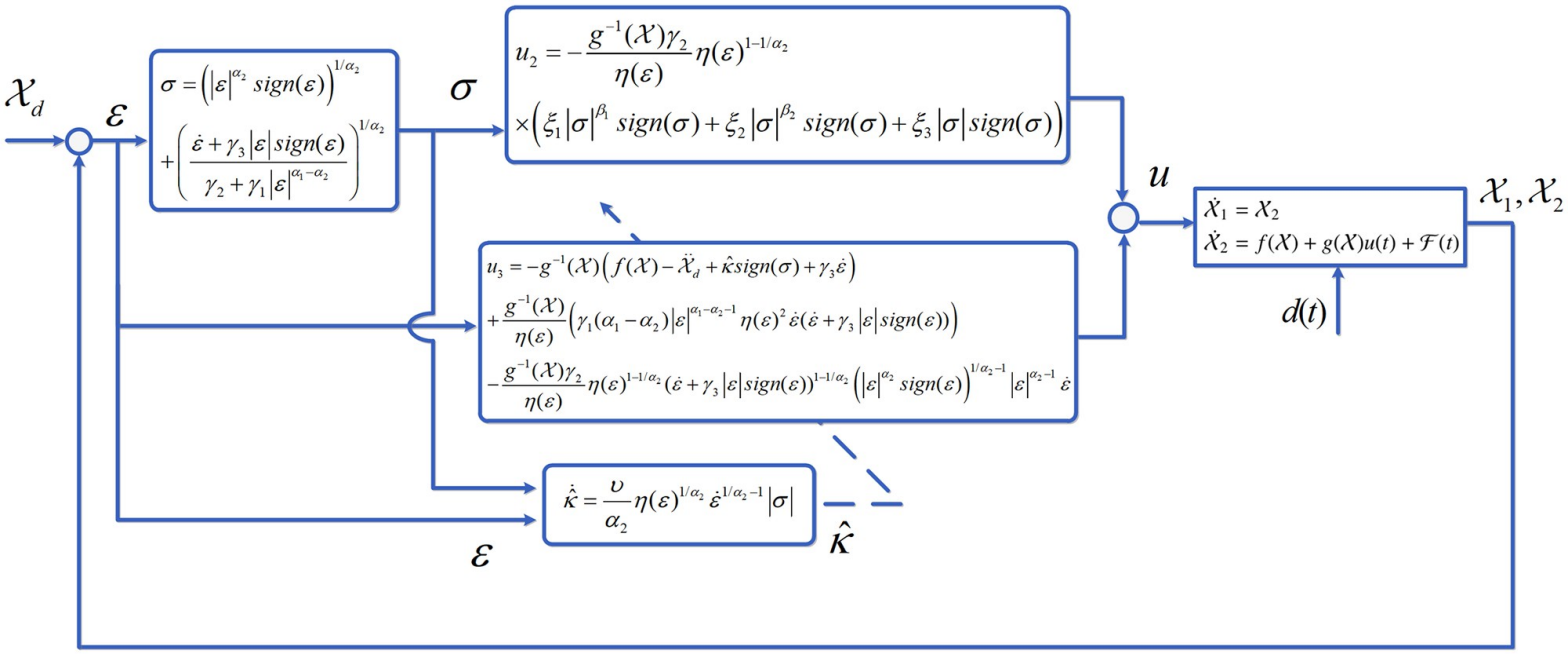
Proposed model diagram.

*Remark 2.* It is stated that state convergence within a fixed-time interval is attained by applying the suggested approach, which integrates the sliding surface, adaptive fixed-time TSM scheme, and adaptive rule, to the nonlinear dynamical system. In Fig 1, the complete developed model is depicted. Furthermore, Lemma 1 suggests that the fixed-time, denoted by $T_{b}$, can be strongly impacted by the selection of parameters, such as *γ*_*i*_ and *ξ*_*i*_. The speed of convergence will be affected when these parameters are tuned. The computer simulation findings are going to be presented in the following section.

## 4. Numerical results

In this section, four cases are given to control the nonlinear uncertain systems with disturbance to analyze the effectiveness of the simulation and validate the suggested scheme. The numerical simulations are intended to demonstrate the performance in the absence and presence of uncertainty, parameter variation, and unknown disturbance, and to be compared with the existing control schemes. The given examples are provided and simulated using MATLAB/Simulink to verify the findings of this study.

### Case 1

In this case, the comparative simulations are shown in the absence of the disturbance. For this analysis, intended trajectory, dynamical system model, and parameters of the nonlinear model are given. Now, we may express the following second-order nonlinear system as [37]:

$$\begin{array}{c} \begin{array}{c} {\dot{X}}_{1}=X_{2},\\ {\dot{X}}_{2}=\left(3+\text{cos}\left(X_{1}\right)\right)\phi -X_{1}^{2}-1.5X_{2}+2\text{sin}\left(X_{2}\right)\\ +\left(3+\text{cos}\left(X_{1}\right)\right)u\left(t\right)+F\left(t\right)+d\left(t\right) \end{array} \end{array}$$ (35)

with the parameters *ϕ* = 2. The parameters of the proposed scheme are given as follows: *γ*_1_ = 100, *γ*_2_ = 30, *γ*_3_ = 30, *ξ*_1_ = 300, *ξ*_2_ = 100, *ξ*_3_ = 30, *α*_1_ = 1.95, *α*_2_ = 0.9, *β*_1_ = 1.5 and *β*_2_ = 0.7. In addition, the initial parameters of $X_{1}$ and $X_{2}$ are provided as $X_{1}\left(0\right)=0.5$ and $X_{2}\left(0\right)=0$. Making sure the system’s trajectories meet with the chosen desired input $X_{d}=\begin{array}{c} 0.6e^{-4t}-1.4e^{-t}+1.45 \end{array}$ is one of the main objectives of the proposed scheme. And constant with adaptive rule is *υ* = 1 and the initial parameter is given as *κ*(0) = 2.

**Fig 2:**
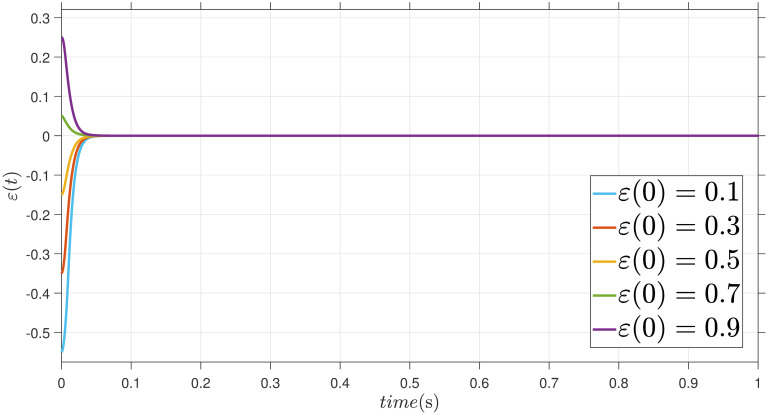
Error *ε* at different initial values.

**Fig 3:**
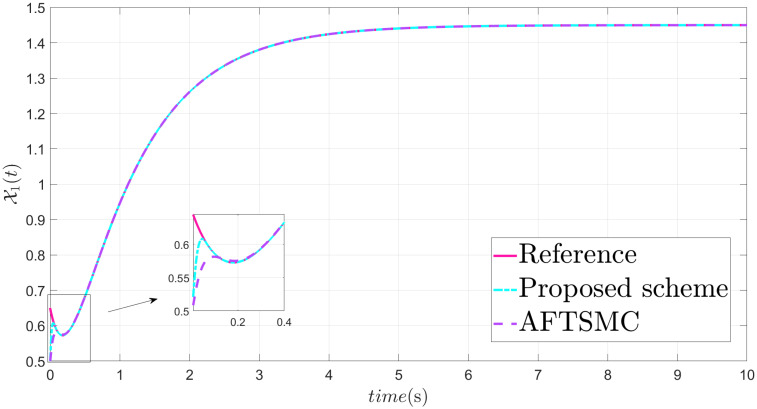
State $X_{1}$ tracking.

**Fig 4:**
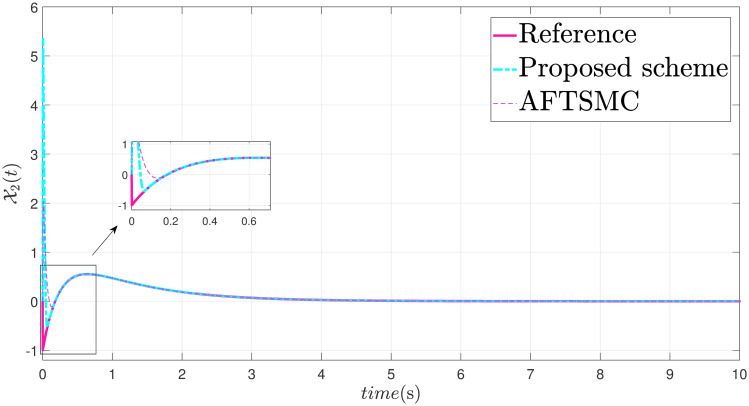
State $X_{2}$ tracking.

**Fig 5:**
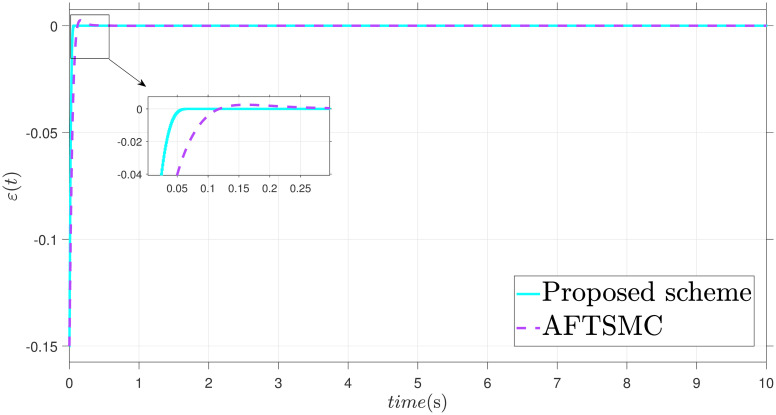
Tracking error *ε*.

**Fig 6:**
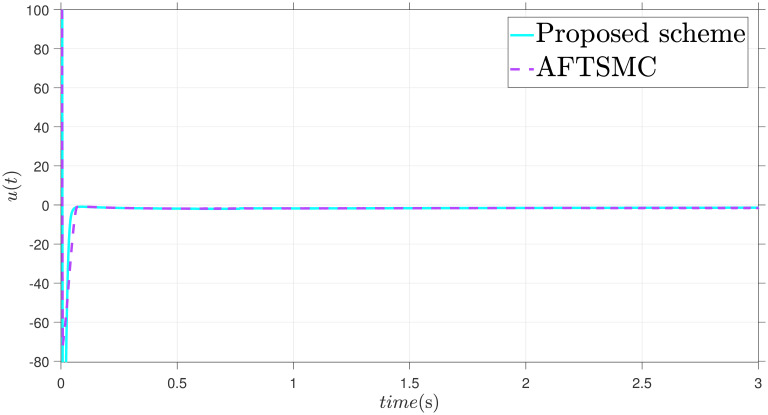
Control input *u*.

The parameters are suitably tuned, and the tracking error performance of the proposed fixed-time scheme at different initial conditions is given in Fig 2. Then, a detailed comparison between the suggested adaptive fixed-time TSM and adaptive finite-time TSM (AFTSMC) [35] is now being performed. It is clear from examining Figs 3–5 that the suggested strategy performs better in terms of tracking, minimum error, and reaches convergence faster. Moreover, Fig 6 depicts the control input and illustrates that the suggested solution results in a lessened chattering problem.

### Case 2

**Fig 7:**
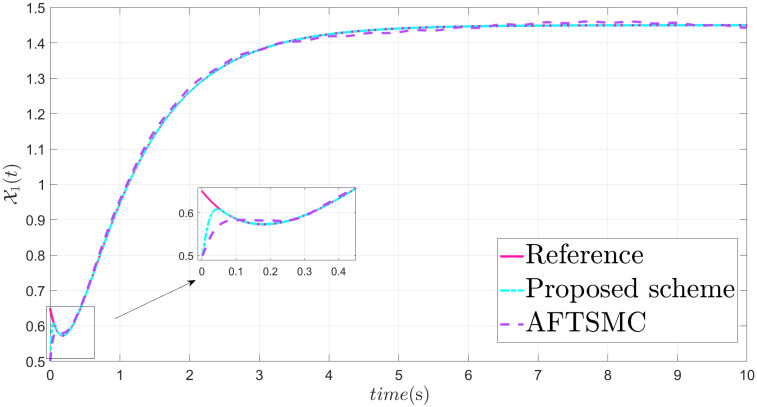
State $X_{1}$ trajectory with disturbance.

**Fig 8:**
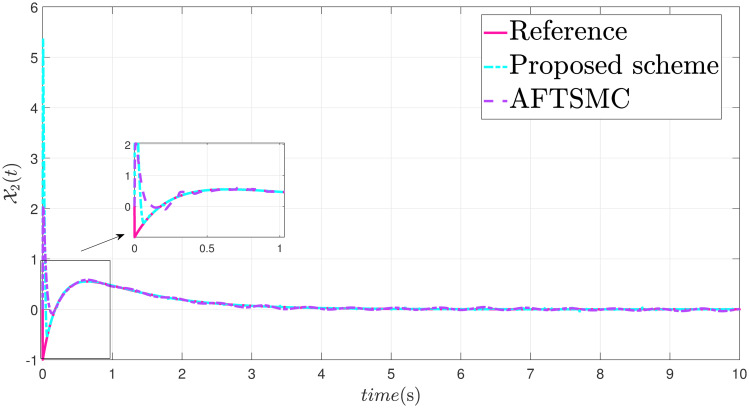
State $X_{2}$ trajectory with disturbance.

**Fig 9:**
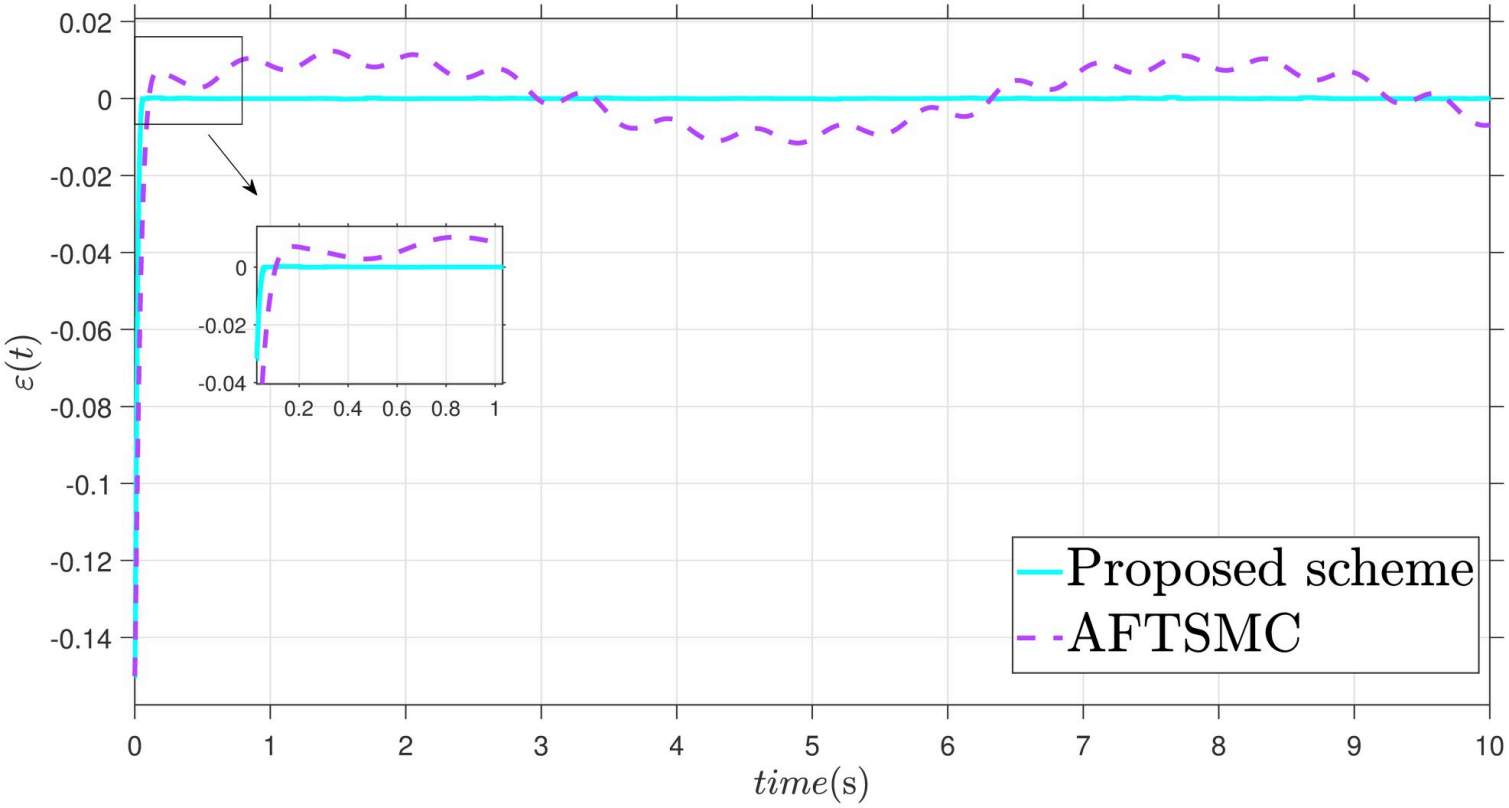
Error *ε* with disturbance.

**Fig 10:**
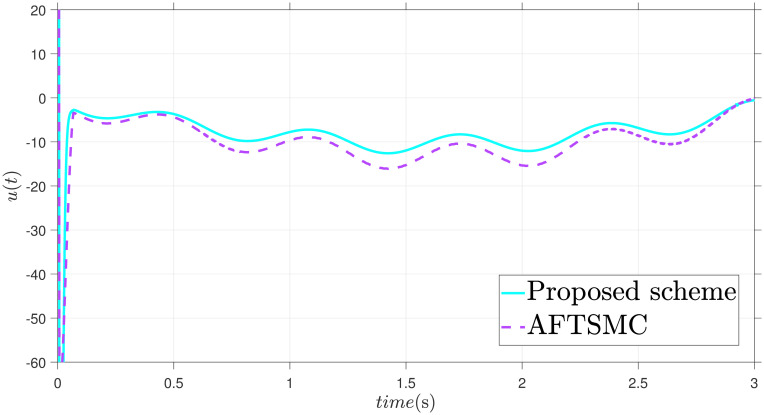
Control input *u* with disturbance.

A comparison with the results of the fast nonsingular sliding mode control scheme [35] is done to highlight the advantages of the suggested adaptive fixed-time TSM method in the face of the unknown external disturbance. The details about the uncertain dynamics and external disturbance are given by $F\left(t\right)=10sin\left({\dot{X}}_{1}\right)$ and *d*(*t*) = 30*sin*(*t*) + 7.5*sin*(10*t*), respectively. Since the goal of this work is to establish an efficient control scheme for second-order nonlinear dynamics, a study is conducted to demonstrate the efficacy of the proposed control approach in comparison with the results provided by [35]. The states tracking, tracking error, and control performance are depicted in Figs 7 to 10.

Figs 7–9 illustrate graphs that show the states $X_{1}$, $X_{2}$, and tracking error *ε*. The satisfactory performance of states tracking has been successfully achieved. We may observe that the proposed and compared schemes rapidly reduce the tracking error to zero. Nonetheless, the control method defined in [35] struggles to accomplish precise tracking, and one can see that the proposed scheme yields swift convergence. Fig 10 shows the control input. The recommended control input *u* is smooth and performs well enough in tracking to effectively reduce external disturbance. Moreover, compared to the control law provided in [35], it is observed that the proposed scheme takes less effort.

### Case 3

This case presents the simulations of the robotic system; therefore, the dynamics of a single-link robotic manipulator are taken into account while uncertainties and outside disturbance are present. The comparative simulations between the proposed scheme and fixed-time TSM (FxTSM) [28] are illustrated. The single-link manipulator dynamics its parameters, desired trajectory, applied uncertain dynamics, and disturbances are given as [38]:

$$\begin{array}{c} {\dot{x}}_{2}=-9.81sin\left(x_{1}\right)-3x_{2}+0.5u+F\left(t\right)+d\left(t\right) \end{array}$$

**Fig 11:**
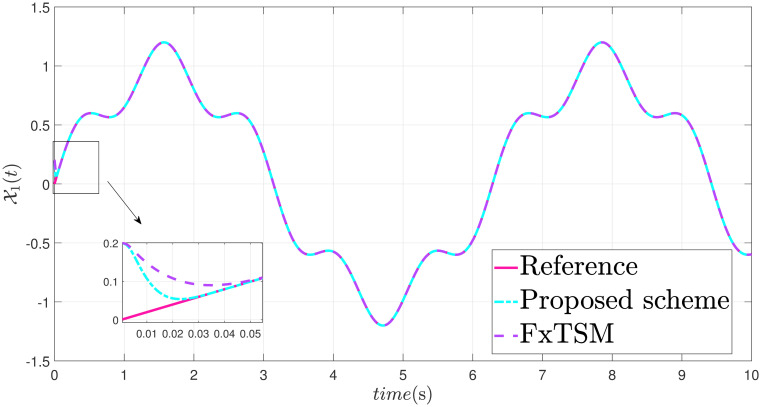
State $X_{1}$ trajectory with disturbance.

**Fig 12:**
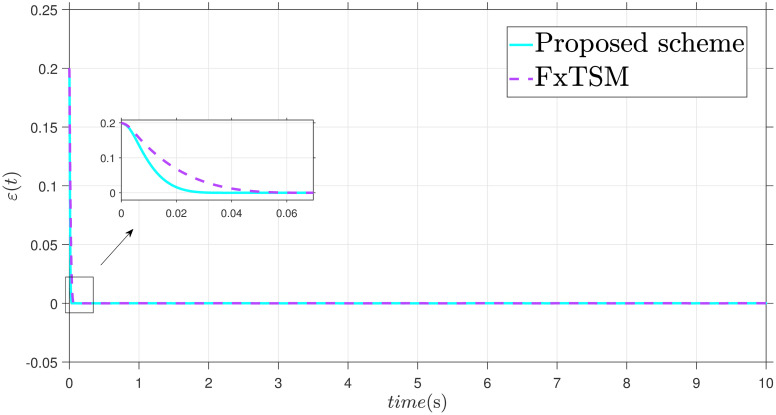
Error *ε* with disturbance.

**Fig 13:**
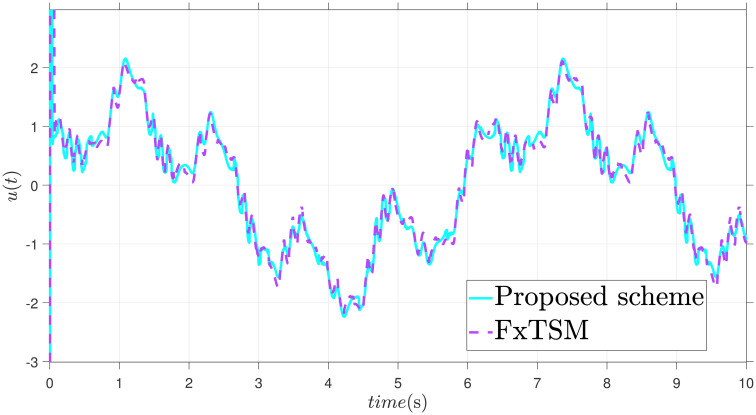
Control input *u* with disturbance.

**Fig 14:**
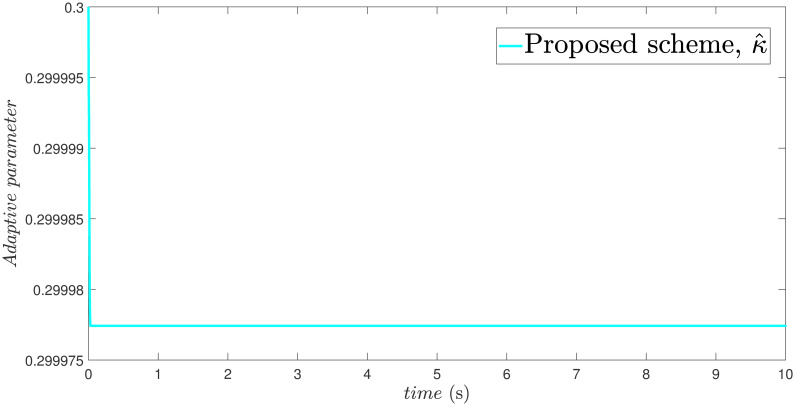
Adaptive parameter estimation $\hat{\kappa }$.

The parameters of the suggested method are selected as: *γ*_1_ = 100, *γ*_2_ = 40, *γ*_3_ = 30, *ξ*_1_ = 300, *ξ*_2_ = 100, *ξ*_3_ = 30, *α*_1_ = 1.95, *α*_2_ = 0.9, *β*_1_ = 1.5 and *β*_2_ = 0.7. $X_{d}=sin\left(t\right)+0.2sin\left(5t\right)$, $F\left(t\right)=sin\left(10X_{2}\right)+cos\left(X_{1}\right)$, *d*(*t*) = 2*sin*(*t*) + 0.5*cos*(10*t*). The initial value is set as $X_{1}\left(0\right)=0.2$. Moreover, adaptive constant is *υ* = 1 and the initial parameter is given as *κ*(0) = 0.3. The performance of the proposed control strategy is comprehensively evaluated through simulations. Figs 11–14 each depict a specific aspect of the system’s response. Fig 11 illustrates the achieved position tracking performance, allowing for graphical assessment of how closely the system’s output follows the desired trajectory. Fig 12 focuses on the tracking error, providing quantitative information about the difference between the reference and actual positions. Fig 13 presents the control torque exerted by the system to achieve the desired motion. Finally, Fig 14 explores the behavior of the adaptive parameters within the controller, offering insights into how they adjust to optimize system performance.

Simulation outcomes from Figs 11–14, provide compelling evidence that the proposed control scheme outperforms the FxTSM approach in terms of tracking accuracy and convergence speed. This reflects the system’s ability to more precisely follow the desired trajectory. Additionally, the proposed scheme achieves this desired state in a short time compared to FxTSM, indicating its efficiency. These combined improvements in tracking performance and convergence characteristics establish the proposed scheme as a more effective solution for the investigated control problem.

### Case 4

**Fig 15:**
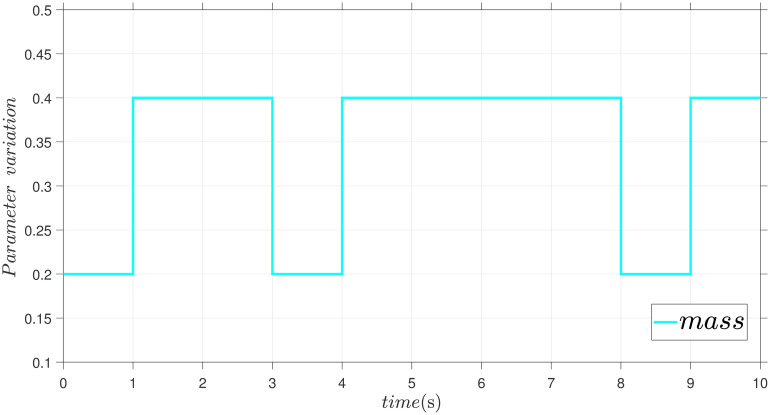
Parameter variation.

**Fig 16:**
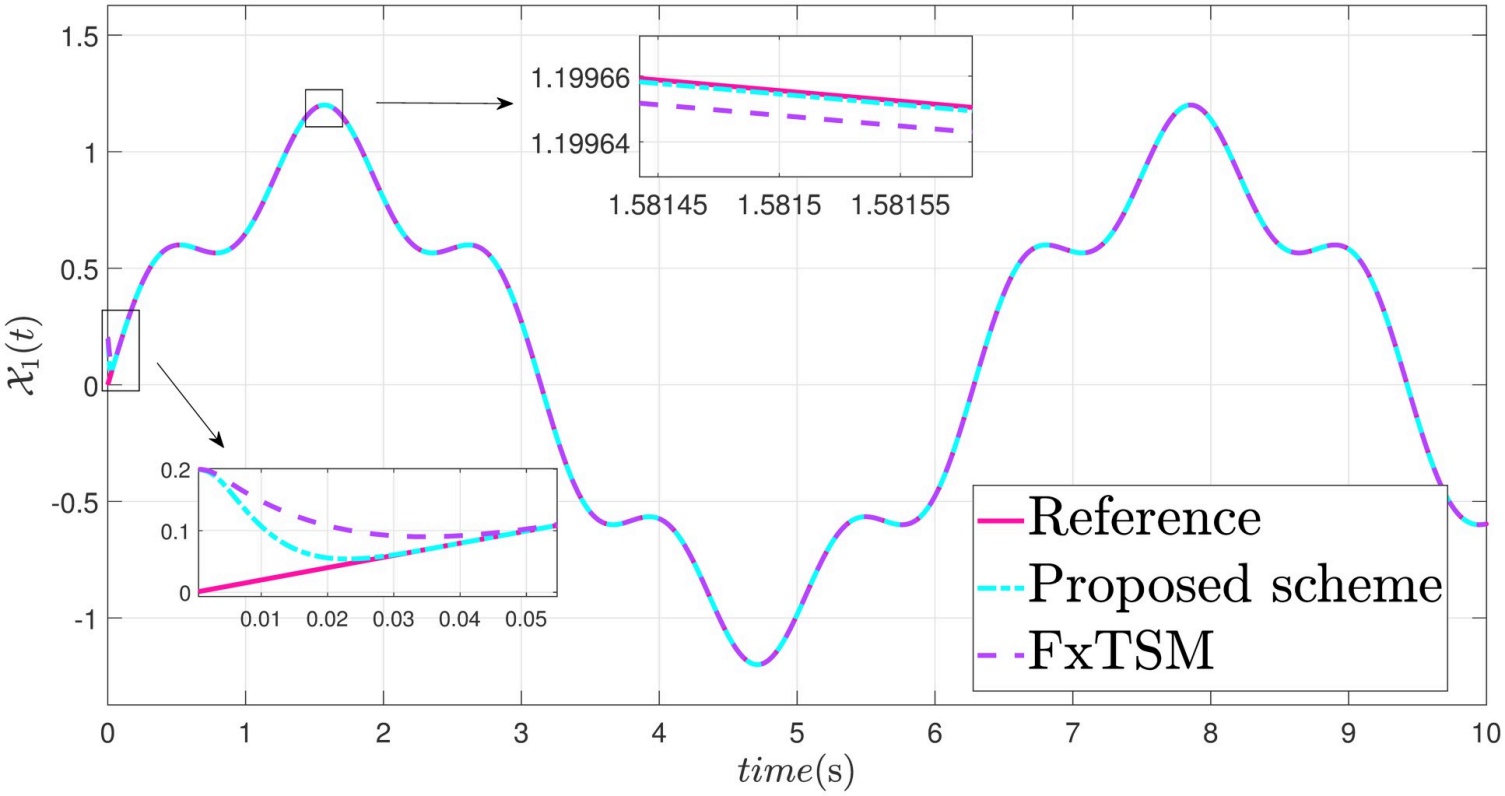
State $X_{1}$ trajectory with parameter variation.

**Fig 17:**
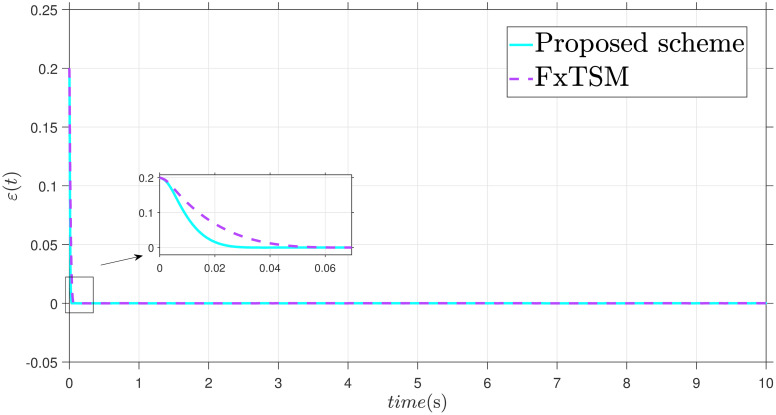
Error *ε* with parameter variation.

**Fig 18:**
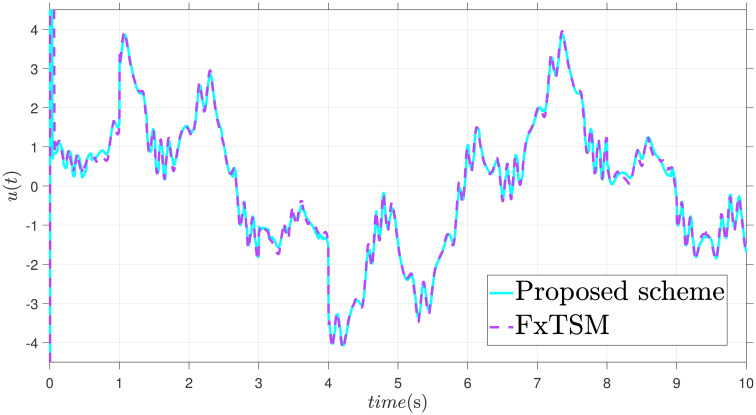
Control input *u* with parameter variation.

This case presents the simulations of the dynamics of a single-link robotic manipulator under the uncertainties, external disturbance and parameter variation. The robustness and adaptiveness of the proposed control approach was rigorously assessed through simulations. Thus, a key aspect of this evaluation involved varying the mass of the robotic manipulator during the simulations. This variation in mass represents a common source of uncertainty in real-world manipulator applications, such as manipulator arms with payloads that can change throughout simulation. The comparative performances between the proposed method and FxTSM under parameter variation (Fig 15), such as position tracking, error, and control torques, are demonstrated in Figs 16–18, respectively.

The simulations show that the controller can continue to work well even when there are unforeseen changes in the dynamics of the system, as seen by its capacity to sustain performance in spite of these mass variations.

## 5. Discussions and analysis

The simulated results of the proposed fixed time TSM method have been presented. This section now offers an in-depth investigation into the limitations given by the recommended controller. This paper conducts a thorough analysis of the limitations related to the recommended controller gain values and stability demonstrations. Furthermore, this theoretical discourse will go further into the possible uses of the suggested methodology in a nonlinear dynamical system. As a result, a nonlinear system has been used to evaluate and validate the suggested scheme’s effectiveness, and the analysis also shows that it performs better than the comparison scheme.

The parameters that have been chosen in compliance with the provided range as the appropriate ones for the suggested method are *γ*_1_ > 0, *γ*_2_ > 0, *γ*_3_ > 0, *ξ*_1_ > 0, *ξ*_2_ > 0, *ξ*_3_ > 0, *α*_1_ > 1, *β*_1_ > 1, and *α*_2_, *β*_2_ ∈ (0, 1). Although the recommended scheme in this instance remains unchanged, ignoring these requirements may cause the system’s closed-loop stability to become unstable. According to the settling time equation, there is an inversely proportional relationship between *γ*_*i*_ and $T_{a}$, as well as between *ξ*_*i*_ and $T_{b}$, except for the directly proportional relationship between *γ*_*i*_, *ζ*_*i*_ and *u*(*t*). The values of *γ*_*i*_ and *ξ*_*i*_ must be adequately adjusted to achieve both closed-loop stability and error convergence within a fixed time. As a result, these values will be a significant element in determining the system’s stability. To some extent, it is possible to choose a reasonable value when details about the precise ranges that each parameter falls within are known. This simplifies the process of determining a suitable value of the proposed control scheme.

## 6. Conclusion

An adaptive fixed-time TSM scheme has been developed to achieve the high states’ trajectory tracking of an uncertain nonlinear dynamical system under external disturbance. The ability to deal with the unknown bounds of system dynamics requires the application of the suggested adaptive scheme. Using this method, the fixed-time TSM scheme demonstrates convergence in a fixed amount of time and provides sufficient trajectory tracking performance. Utilizing the proposed adaptive fixed-time TSM for a nonlinear system with unknown disturbance, we demonstrate the effectiveness of the developed technique by utilizing one of its applications on a second-order system. The results indicate that, in comparison to the AFTSMC and FxTSM approaches, the adaptive fixed-time TSM method performs better in terms of quicker response times, lower tracking errors, and improved control over unknown dynamics. In addition, the tracking performances are given of the proposed *ε*_*rms*_ = 0.0043 in comparison with *ε*_*rms*_ = 0.0101 of AFTSMC in case 2, and the proposed *ε*_*rms*_ = 0.0054 in comparison with *ε*_*rms*_ = 0.0066 of FxTSM in case 3. For future work, this scheme can be modified with the fractional-order scheme and applied to the higher-order system.
